# Boron Induces Early Matrix Mineralization via Calcium Deposition and Elevation of Alkaline Phosphatase Activity in Differentiated Rat Bone Marrow Mesenchymal Stem Cells 

**DOI:** 10.22074/cellj.2016.3988

**Published:** 2016-04-04

**Authors:** Bent-al-hoda Movahedi Najafabadi, Mohammad Hussein Abnosi

**Affiliations:** Department of Biology, Faculty of Sciences, Arak University, Arak, Iran

**Keywords:** Boric Acid, Mesenchymal Stem Cells, Morphology, Osteoblasts

## Abstract

**Objective:**

Boron (B) is essential for plant development and might be an essential micronutrient for animals and humans. This study was conducted to characterize the impact of boric acid (BA) on the cellular and molecular nature of differentiated rat bone marrow mesenchymal stem cells (BMSCs).

**Materials and Methods:**

In this experimental study, BMSCs were extracted and expanded to the 3rdpassage, then cultured in Dulbecco’s Modified Eagle’s Medium (DMEM) complemented with osteogenic media as well as 6 ng/ml and 6 µg/ml of BA. After 5, 10, 15 and 21 days the viability and the level of mineralization was determined using MTT assay and alizarin red respectively. In addition, the morphology, nuclear diameter and cytoplasmic area of the cells were studied with the help of fluorescent dye. The concentration of calcium, activity of alanine transaminase (ALT), aspartate transaminase (AST), lactate dehydrogenase (LDH) and alkaline phosphatase (ALP) as well as sodium and potassium levels were also evaluated using commercial kits and a flame photometer respectively.

**Results:**

Although 6 µg/ml of BA was found to be toxic, a concentration of 6 ng/ml increased the osteogenic ability of the cell significantly throughout the treatment. In addition it was observed that B treatment caused the early induction of matrix mineralization compared to controls.

**Conclusion:**

Although more investigation is required, we suggest the prescription of a very low concentration of B in the form of BA or foods containing BA, in groups at high risk of osteoporosis or in the case of bone fracture.

## Introduction

Boron (B), a chemical member of group 3A is the fifth element in Mendeleev’s periodic table and a semiconductor element with metal and non-metals properties. It is found in considerable quantity (range 20-43 μg/g) in fruits and vegetables such as apple, grapes, celery, tomato, soy meal, dried fruits, legumes, nuts, almond, avocado and banana (1, 2). B can be completely absorbed by the gastrointestinal tract from drinking water and plantderived foods, and circulates in the blood as boric acid (BA) (3). The first study which suggested that B is beneficial and may be essential for animals, showed that B can improve bone calcification in chicks fed on a diet partially deficient in vitamin D (4). B affects metabolic regulation via the formation of complexes with a variety of substrates or reactant compounds having hydroxyl groups in favorable positions (5). In recent years, the effect of B on osteogenesis and the maintenance of bone have been examined *in vivo* (6). B may interact with steroid hormones (7), and prevent calcium loss and bone demineralization (8). It has been shown that B supplementation in periand postmenopausal women reduces urinary calcium and magnesium excretion as well as increasing serum levels of estradiol and calcium absorption (9).

Recently it was shown that the mineralization of bone-related cells may be affected by B. Hakki et al. (10) using MC3T3-E1 (a mouse C57/B6 calvarial osteoblastic cell line), investigated the effects of BA on cell proliferation, mineralization, and mRNA expression of mineralization associated genes. They found that 1 or 10 ng/ml compared to 0 and 0.1 ng/ml increased mineralized nodule formation and mRNA expression of type 1 collagen, osteopontin, bone sialoprotein, osteocalcin, and RunX. In addition they found that the BA supplementation increases bone morphogenetic proteins 4, 6, and 7. Ying et al. (11) demonstrated that proliferation of human bone marrow mesenchymal stem cells (BMSCs) was not affected by treatment with 10 and 100 ng/ml of BA. However, these concentrations did cause elevation of alkaline phosphatase (ALP) activity and increased mRNA expression of ALP, osteocalcin, collagen type I, as well as bone morphogenetic proteins 7. In a previous report we showed that the viability of rat BMSCs was not affected by short exposure (12 hours) to low dose BA (6 ng/ml), but increased exposure time (24 and 36 hours) would decrease metabolic activity and viability. In addition, we showed that 6 ng/ml of BA caused a significant increase in ALP activity and total calcium concentration in BMSCs (12). Very little is known about the biological effects of B exposure in animal cells, especially which concentration has a positive effect on mesenchymal stem cell differentiation to osteoblasts. Based on the above findings, the present study was designed to determine the morphological and biochemical changes in osteogenic differentiated BMSCs treated with different doses of BA *in vitro*.

## Materials and Methods

### Isolation and expansion of rat bone marrow mesenchymal stem cells

In this experimental study, Wistar rats (6-8 weeks old) were purchased from the Pastor Institute (Tehran, Iran) and kept in the animal house of Arak University under standard conditions of light and food. Following sacrifice by excessive chloroform (Merck, Germany) inhalation the animals’ tibia and femurs were removed and cleaned from adherent soft tissue. The two ends of the bones were then cut off and bone marrow flushed out using 2 ml of Dulbecco’s Modified Eagles Medium (DMEM, Gibco, Germany) supplemented with 15% fetal bovine serum (FBS, Gibco, Germany) and penicillin-streptomycin (Gibco, Germany). Bone marrow content was centrifuged at 2500 rpm for 5 minutes and re-suspended in 5 ml DMEM containing 15% FBS and antibiotics then plated in culture flasks and incubated at 37˚C in an atmosphere containing 5% CO2. The first medium replacement was performed one day after culture initiation, after which the medium was changed twice a week till the bottom of the flask was covered with cells (i.e. until confluency). The cells were trypsinized trypsin-Ethylenediaminetetraacetic acid (EDTA, Gibco, Germany) and passed to another culture flask as the first passage. The cultures were then expanded through two additional subcultures after which they were used for further investigation (12). All the procedures were approved by the experimental animal Ethics Committee of Arak University.

### Osteogenic induction

Mineralization was induced on confluent monolayers of cells by the addition of DMEM containing 15% (v/v) FBS, streptomycin-penicillin and osteogenic supplements (1 mM sodium glycerophosphate, 50 μg/ml L-ascorbate and 10-8 M dexamethasone). All the chemicals were purchased from Sigma-Aldrich, America unless otherwise stated. The culture flasks were then incubated at 37˚C with 5% CO_2_ and their medium was changed every 3 days for 21 day (13).

### Exposure to boric acid

BA (Merck, Germany) was used to make different concentrations of B since in humans and animals B is transported as BA. BA was prepared as a stock solution of 6 mg/ml, and the pH of the solution was adjusted to 7.3 which is appropriate for cell culture.

### Cell viability assays

The viability test on control and treated cells was carried out using an MTT assay in an ELISA plate. In live cells mitochondrial succinate dehydrogenase converts the yellow colored tetrazolium salts into violet crystals of formazan after 4 hours of incubation. At the end of the incubation period 100 μl of dimethyl sulfoxide (DMSO) was added to each well of the plate and the formazan crystals were extracted following incubation for 30 minutes at room temperature. The solution was transferred into another well and the absorbance measured at 505 nm using an automated microplate reader (SCO diagnostic, Germany) (13).

### Analysis of morphological changes

Following BA treatment in osteogenic media for 5, 10, 15 and 21 days at room temperature the nuclear morphology of the cells was studied after 5 minutes incubation in the dark using Hoechst 33342 staining. The diameter of the cells was measured in μm using Motic Image software (Micro optical group company version 1.2). Hoechst is a fluorescent dye which penetrates the cells through the intact plasma membrane and stains the DNA so changes in nuclear morphology, such as chromatin condensation and fragmentation, can be investigated (14). In addition, the morphology of the cell cytoplasm was investigated using acridine orange fluorescent dye. The cells after staining were washed twice with phosphate-buffered saline (PBS), examined and immediately photographed under an invert fluorescence microscope (Olympus, IX70) equipped with a camera.

### Detection and quantification of mineralization

Treated and control cells were cultured in 6-well plates for 5, 10, 15 and 21 days, then washed with PBS and fixed in 10% (v/v) formaldehyde at room temperature for 15 minutes. To carry out the staining procedure the cells were washed twice with an excess of dH_2_O then 1 ml of 40 mM alizarin red solution (ARS, pH=4.1) was added per well. After that the plates were incubated at room temperature for 20 minutes with gentle shaking, the excess of dye was poured off and the plates were washed four times with dH_2_O. Stained cells were investigated under a light inverted microscope and photographed. To quantify the level of absorbed alizarin red, 800 μl of 10% acetic acid (v/v) was added to each well, and the plate was incubated at room temperature for 30 minutes with gentle shaking. Then the loosely attached cells were scraped from the plate with a cell scraper and transferred to a 1.5 ml micro-centrifuge tube. After vortexing for 30 seconds, the slurry was overlaid with 500 μl mineral oil, heated at 85˚C for 10 minutes, and then kept on ice for 5minutes. The slurry was then centrifuged at 20,000 g for 15 minutes and 500 μl of the supernatant was transferred to a new micro-centrifuge tube and 200 μl of 10% ammonium hydroxide (v/v) was added to neutralize the acid. An aliquot of the supernatant (100 μl) was read in triplicate at 405 nm in a microplate reader (SCO diagnostic, Germany) and quantified against a standard graph.

To prepare the alizarin red standards graph, working ARS (40 mM) was diluted 20 times with a mixture of 5:2 of 10% acetic acid and 10% ammonium to give a concentration of 2000 μM. Different standard solution ranging from 2000 to 31.3 μM was prepared and the absorption taken at 450nm using a microplate reader. The concentration of the unknown samples was calculated using the linear formula Y=0.099X+0.101 with R^2^=0.997 where Y is the absorbance and X is the concentration (mM) of alizarin red (14).

### Preparation of cell extract

Control and osteogenic cells treated with 6 ng/ml and 6 μg/ml of BA for 5, 10, 15 and 21 days were washed with Tris-HCl-NaCl buffer. Then the loosely attached cells were scraped off the plate with the cell scraper, ground in liquid nitrogen and the cell content extracted with Tris-HCl buffer followed by centrifugation at 12000g for 10minutes. The total protein content of each sample was determined by the Lowry method, using bovine serum albumin (BSA) as standard. The standard graph was plotted and the concentration of the unknown protein samples was calculated using the linear formula Y=0.003X+0.036 with R^2^=0.998 where Y is the absorbance and X is the concentration (μg) of the protein in each sample.

### Determination of alkaline phosphatase activity

ALP activity was determined in a protein lysate base on equal amounts of protein using para-nitrophenylphosphate (pNPP) as substrate according to the kit instructions (Parsazmon, Iran). Absorbance at 405 nm was measured using a spectrophotometer (T80^+^PG instrument Ltd, England).

### Determination of transaminases and lactate dehydrogenase activity

Alanine transaminase (ALT), aspartate transaminase (AST) and lactate dehydrogenase (LDH) activity was determined in a protein lysate base on an equal amount of protein according to the kit instruction (Parsazmon, Iran). Absorbance at 340 nm was measured using a spectrophotometer (T80^+^PG instrument Ltd, England).

### Calcium concentration

Control and treated cells were washed twice with PBS then incubated for 24 hours with 50 μl of 0.5 N HCl to dissolve the calcium content. The amount of calcium was determined using a commercial kit (Parsazmon, Iran) and the developed color was measured at 575 nm using a spectrophotometer (T80^+^PG instrument Ltd, England). Using different concentrations of calcium chloride a standard graph was prepared and the concentration of unknown samples was calculated using the linear formula Y=0.0763X-0.0039 with R^2^=0.998 where Y is the absorbance and X is the concentration (mg/dl) of calcium.

### Na^+^ and K^+^ concentration

The levels of Na^+^ and K^+^, were determined using a flame photometer (Model PFP7, England) with filters for sodium and potassium. Using different concentrations of sodium and potassium chloride a standard graph was plotted and the concentration of sodium and potassium in the unknown samples was calculated using the linear formula Y=0.0173X+0.021 with R^2^=0.992 and Y=0.4803X+0.0223 with R^2^=0.996 respectively. In the formula, Y is the absorbance and X is the concentration (μg/ml) of each ion.

### Statistical analysis

Statistical evaluation of the data was performed in SPSS (version 16, Sun Microsystems Inc., America) using one-way ANOVA and the Tukey test. Results are shown as mean ± SD, and P<0.05 was accepted as the minimum level of significance.

## Results

### Effect of boric acid on cell viability

**Fig.1:**
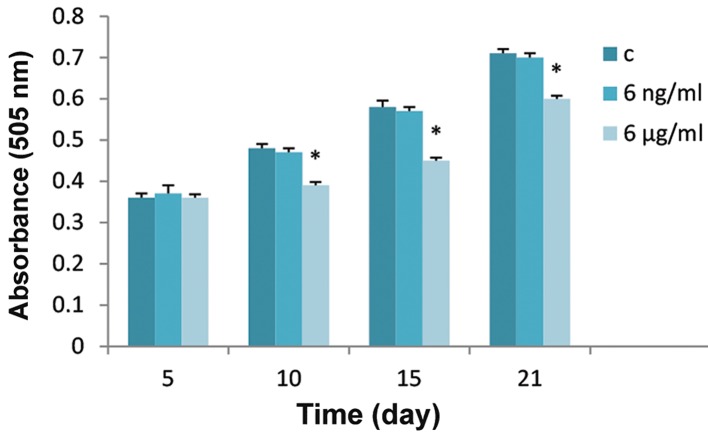
Cell viability of BMSCs after 5, 10, 15 and 21 days of treatment with 6 ng/ml and 6 μg/ml of BA in osteogenic media. "c" in the legend stands for control group where no treatment with BA has been received. The data are represented as mean ± SD (One-way ANOVA, Tukey test, *P<0.05). BMSCs; Bone marrow mesenchymal stem cells and BA; Boric acid.

Cell viability assays (Fig.1) showed that 6 μg/ml of BA significantly decreased the viability of BMSCs under osteogenic differentiation on days 10, 15 and 21 (P<0.001) compared with controls, but no effect was observed on day 5. The lower dose of BA (6 ng/ml) showed no significant effect (P>0.05) on the viability of the cells at any of the treatment periods.

### Boric acid induced morphological changes in differentiated bone marrow mesenchymal stem cells

**Fig.2:**
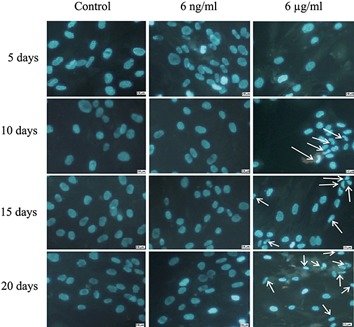
Fluorescent micrograph images of BMSCs stained with Hoechst, after 5, 10, 15 and 21 days of incubation in osteogenic media treated with 0, 6 ng/ml and 6 μg/ml of BA. Nuclear condensation and deformation (arrows) of cells was observed after treatment with 6 μg/ml of BA (×40 magnification). BMSCs; Bone marrow mesenchymal stem cells and BA; Boric acid.

**Fig.3:**
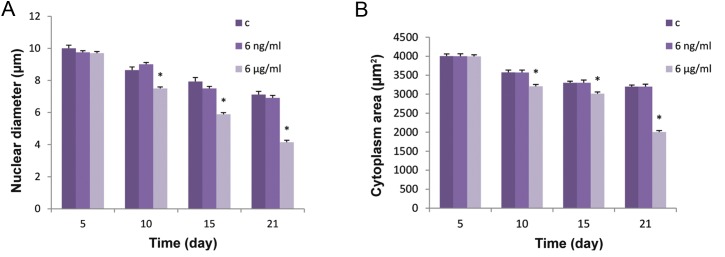
Morphological changes in BMSCs incubated in osteogenic media after 5, 10, 15 and 21 days of treatment with 0, 6 ng/ml and 6 μg/ ml of BA. A. Diameter of nucleus (μM) and B. Area of cytoplasm (μm^2^). "c" in the legend stands for control group where no treatment with BA has been received. Values are means ± SD (One-way ANOVA, Tukey test, *P<0.05). BMSCs; Bone marrow mesenchymal stem cells and BA; Boric acid.

**Fig.4:**
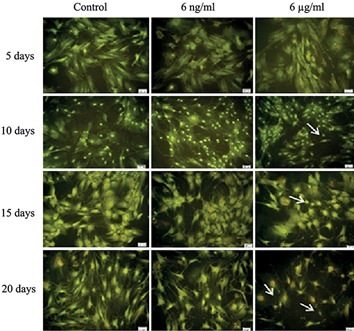
Fluorescent micrograph images of BMSCs stained with acridine orange, after 5, 10, 15 and 21 days of incubation in osteogenic media treated with 0, 6 ng/ml and 6 μg/ml of BA. Shrinkage and complete disappearance of the cytoplasm in some cells (arrows) was observed after treatment with 6 μg/ml of BA (×20 magnification). BMSCs; Bone marrow mesenchymal stem cells and BA; Boric acid.

Morphological study of the nuclei in differentiated mesenchymal stem cells treated with 6 μg/ml of BA after 10, 15 and 21 days showed chromatin condensation and nuclear deformation (Fig.2) as well as a significant reduction (P<0.001) in the diameter of the nuclei (Fig.3A). In addition a significant (P<0.05) reduction in the area of cytoplasm (Fig.3B) was observed compared to controls and 6ng/ml treated cells. It can also be seen that BA at 6μg/ml caused remarkable changes in the morphology of the cytoplasm (Fig.4) such as the shrinkage and complete disappearance of cytoplasm in some cells.

### Mineralization analysis base

**Fig.5:**
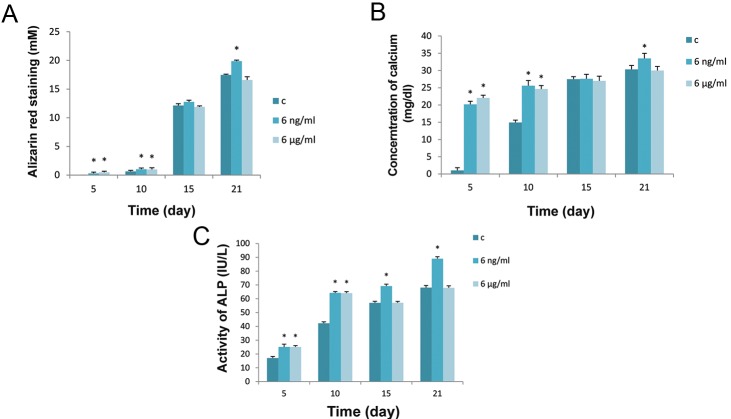
Differentiation analysis of BMSCs cultured in osteogenic medium for different periods and in the presence of BA based on A. Quantitative alizarin red staining (mM), B. Cell matrix Ca^2+^ concentration (mg/dl) and C. ALP activity (IU/L). "c" in the legend stands for control group where no treatment with BA has been received. Values are means ± SD (One-way ANOVA, Tukey test, *P<0.05). BMSCs; Bone marrow mesenchymal stem cells, BA; Boric acid and ALP; Alkaline phosphatase.

**Fig.6:**
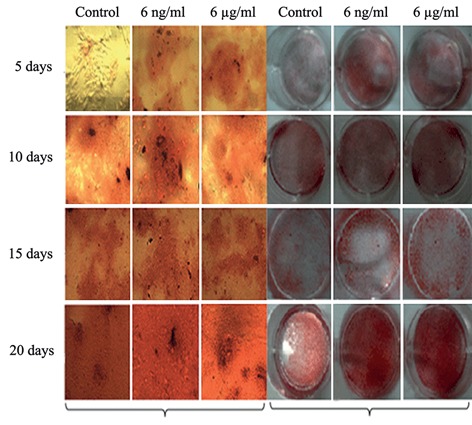
Alizarin red staining of mineralized matrix was performed for BMSCs after 5, 10, 15 and 21 days of incubation in osteogenic media treated with 0, 6 ng/ml and 6 μg/ml of BA, A. Micrograph images (×20 magnification) and B. Macrograph i mages (photographed by digital camera). BMSCs; Bone marrow mesenchymal stem cells and BA; Boric acid.

Mineralization of BMSCs in the presence of different concentrations of BA starts at day 5, where quantitative analysis of alizarin red and calcium concentrations showed a significant increase (P<0.05) compared to controls (Fig.5A, B). In the control group mineralization in the absence of BA mainly starts at day 10 and continues up to days 15 and 21.The presence of BA makes a significant difference by day 10 compared to controls, but reaches the same level by day 15. Mineralization increased significantly by day 21 in the 6 ng/ml of BA group compared to the controls and the 6 μg/ml treated group (Figes.5, 6). Under osteogenic differentiation, ALP activity in control cells is minimal on day 5 and reaches its maximum level on day 21 (Fig.5C). Treatment of the cells with 6 ng/ml and 6 μg/ml of BA caused a significant increase (P<0.05) in the activity of ALP on days 5 and 10 (Fig.5C). By days 15 and 21 a significant increase in ALP activity is seen only in the 6 ng/ml treated cells compared to the control and 6μg/ml treated cells (Fig.5C). Microscopic observation also confirmed the quantitative analysis of alizarin red and calcium concentrations (Fig.6A, B).

### Metabolic activity of the cells

**Fig.7:**
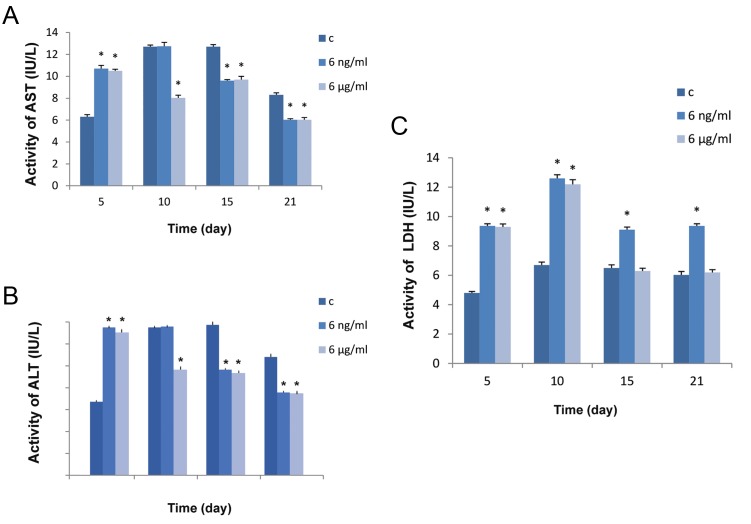
Enzyme activity of BMSCs in osteogenic media after 5, 10, 15 and 21 days of incubation and treatment with 0, 6 ng/ml and 6 μg/ml of BA, A. Activity of AST, B. Activity of ALT and C. Activity of LDH. "c" in the legend stands for control group where no treatment with BA has been received. Values are means ± SD (One-way ANOVA, Tukey test, *P<0.05). BMSCs; Bone marrow mesenchymal stem cells, BA; Boric acid, ALP; Alkaline phosphatase, AST; Aspartate transaminase, ALT; Alanine transaminase and LDH; Lactate dehydrogenase

Maximum activity of transaminase enzymes (ALT and AST) in the BMSCs in differentiation media was observed on days 10 and 15. On one hand, it was observed that the treatment of cells with BA caused a significant decrease in the activity of the transaminases in days 10, 15 and 21, especially with a concentration of 6 μg/ml compared to controls (Fig.7A, B). On the other hand, compared to controls, treatment of the cells with BA significantly increased (P<0.05) ALT and AST activity on day 5 (Fig.7A, B).

From day 10 onwards LDH activity reached a steady state in the control group, whereas by days 5 and 10 BA treatment had caused a significant increase in the activity of this enzyme. The activity of this enzyme still showed a significant increase (P<0.05) on days 15 and 21 with treatment at the lower dose i.e. 6 ng/ml of BA (Fig.7C).

### Na^+^ and K^+^ concentration

**Fig.8:**
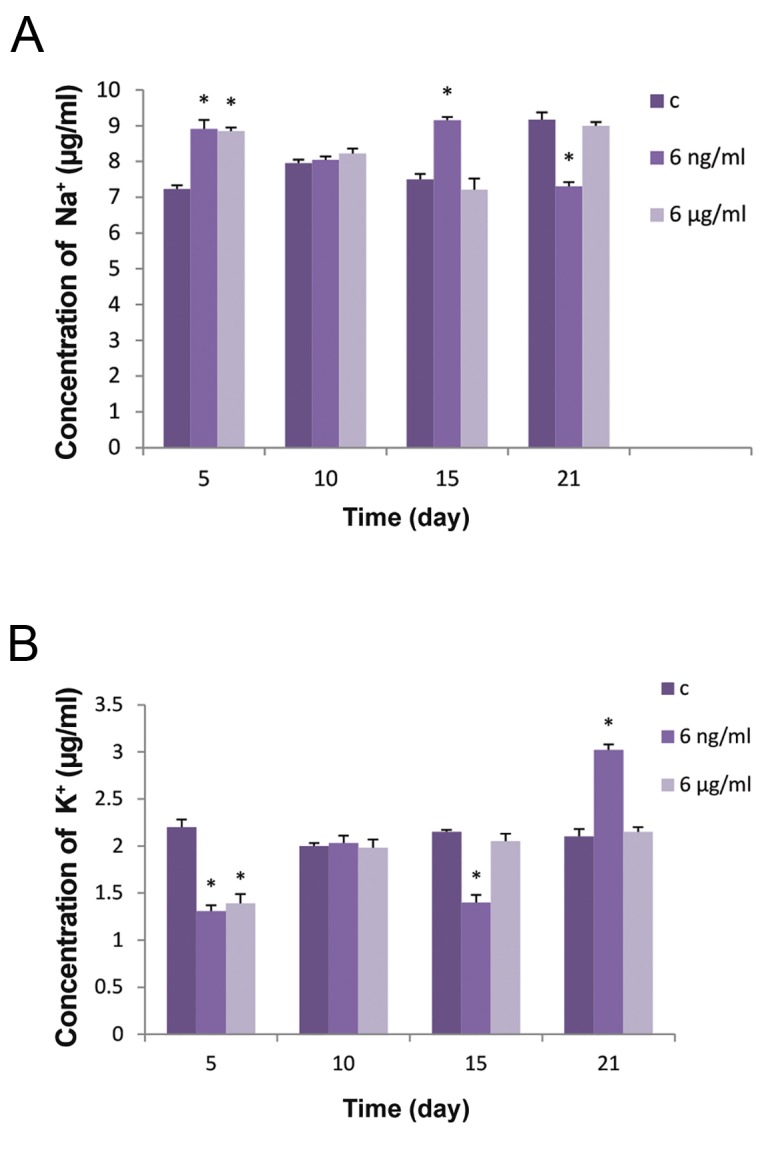
Effect of BA on the Na^+^ and K^+^ concentration of BMSCs after 5, 10, 15 and 21 days of incubation in osteogenic media treated with 0, 6 ng/ml and 6 μg/ml of BA. A. Concentration of sodium (μg/ml) and B. Concentration of potassium (μg/ml). "c" in the legend stands for control group where no treatment with BA has been received. Values are means ± SD (One-way ANOVA, Tukey test, *P<0.05). BA; Boric acid and BMSCs; Bone marrow mesenchymal stem cells.

In the absence of BA, the Na^+^ concentration of BMSCs after osteogenic induction was steady from days 5 to 15, then increase by day 21. Treatment with 6 ng/ml of BA caused a significant increase in the sodium level on day 5 and day 15, whereas a significant decrease was observed on day 21 (Fig.8A). The concentration of K^+^ remained in a steady state from day 5 to 21 in the control group. Treatment of the cells with 6 ng/ml and 6 μg/ml of BA significantly reduced the concentration of potassium by day 5. The effect of 6 μg/ml of BA on the K^+^ level had disappeared by day 10 and there was no significant difference between treated cells and controls at days 15 and 21 (Fig.8B). In contrast, 6ng/ml of BA caused a significant decrease in K^+^ on day 15 and a significant increase on day 21. A surprising result was observed on day 10, where cells treated with BA showed no differences in cell sodium and potassium levels compared to control cells.

## Discussion

B is a micronutrient in plants and may be essential for animal growth and development. In our previous study we showed that 6 ng/ml of BA reduced metabolic activity in BMSCs, but increased ALP activity and total calcium concentration which might have positive effects on osteogenic differentiation (12). For this reason in the present study we investigated the effect of low doses (6 ng and 6 μg/ml) of BA on the differentiation of BMSCs to osteoblasts and attempt to characterize the cellular and molecular nature of differentiated BMSCs.

In the present study we found that the cell viability of differentiated BMSCs was not different from control group when treated with 6 ng/ml of BA, whereas 6 μg/ml of BA could inhibit viability at days 10, 15, and 21. In the previous study we showed that the 6 ng/ml and 6 μg/ml of BA after 24 and 36 hours would cause a reduction in the cell viability of BMSCs (12). As the present study showed no effect of 6 ng/ml BA on the viability of differentiated BMSCs, it might be concluded that osteoblasts are more resistant to B than BMSCs, a result confirmed in a study carried out by Hakki et al. (10). They found that the proliferation of preosteoblasts (MC3T3-E1) was no different from the control group when treated with BA at a concentration of 1, 10, 100 and 1000 ng/ml for 72 hours, and only a slight reduction was observed on days 5 and 14. However, in the same report they claim that only concentrations of 1 and 10 ng/ml is suitable for mineralization in these cells and warn that the higher concentrations might have a negative impact. BMSCs, used in our study, can be found in the bone marrow and are considered as cellular backup for generation of bone related cells such as osteoblasts. In contrast, the study by Hakki et al. (10) used MC3T3-E1 which is an osteoblast precursor cell line derived from Mus musculus (mouse) calvaria, therefore the slight differences between our results and theirs might be due to differences in the origin of the cell lines.

The differentiation of BMSCs to osteoblasts is a key point in the homeostasis of bone as it causes the deposition of calcium to balance mineralization and prevent osteoporosis. For this reason, investigation of the effect of B is necessary to understand the biochemical nature of the mechanisms which are involved. Some reports have indicated the protective effect of B derivatives, such as NaB accompanied by Me2SO, on human tooth germ stem cells which is a cell mesenchymal in nature (15). Although B may be an inhibitor of some metabolic enzymes (16), based on Ying et al. (11) and that of Abnosi and Movahedi (12), there are concentrations of B which might have positive effects on viability and some of the characteristic features of BMSCs. Therefore in the following we discuss the concentration of B shown to have effects on the biochemical mechanisms of differentiated BMSCs.

We found that 6 μg/ml of BA after 10, 15 and 21 days of treatment caused chromatin condensation and nuclear deformation as well as shrinkage of the cytoplasm, factors which together might be considered as having a negative impact due to interference of B with the cell cytoskeleton assembly. Although BA can cause oxidative stress in plant systems (17), it has been reported that low doses of various B compounds in animal systems can increase antioxidant capacity by increasing the activity of enzymes such as superoxide dismutase, catalase, glutathione peroxidase, glutathione-S-transferase, glutathione reductases and Glucose-6-phosphate dehydrogenase (18). Therefore, the reason for the morphological changes caused by 6μg/ml of B might not be due to oxidative stress, since this concentration is not high enough to be the main cause of protein assembly inhibition, but a matter requiring further investigation. However, our results showed that 6 ng/ml BA did not change the morphological state of differentiated BMSCs, thus this concentration of B is nontoxic with respect to viability and morphology.

Our finding showed that 6 μg/ml BA caused no change in the level of mineralization based on quantitative alizarin red, calcium concentration and ALP activity after 15 and 21days of treatment compared to controls. Thus in addition to its toxic effect on viability, 6 μg/ml BA was not effective in promoting differentiation, whereas a significant increase in these factors was observed with 6 ng/ml of BA. Nzietchueng et al. (19) found that B affects the turnover of extracellular matrix via elevation of TNF-alpha release and activity of trypsin-like enzymes such as collagenase and cathepsin D in fibroblasts and thus may cause the improvement of wound healing. Benderdour et al. (20) also demonstrated that BA solution improved wound healing through action on the extracellular matrix, *in vitro*. Our results and those of others show that B is very helpful in the modulation of matrix formation, but the exact cellular mechanism is not clear.

It is well known that hydroxyapatite precipitation takes place at alkaline pH and therefore the proton ion has to be taken out of the osteoblasts (21). The presence of the Na^+^/H+ exchange pump on the cell membrane causes the exchange of Na^+^ in for H+ out to increase the pH and make the internal environment alkaline (22). In addition, it has been shown that the NaBC1 (a homolog of AtBor1 in plants) transporter is present in animal cells which in absence of B transport Na^+^ and OH^-^ (H+), and in presence of borate functions as an electrogenic voltage regulator and transporter of Na^+^ coupled with B(OH)_4_^-^ as co-transporter (23). In this way it helps neutralize the acidic pH inside the cells. In addition, Park et al. (24), showed that a low concentration of B causes the activation of the MAPK pathway which plays an important role in the bone morphogenesis (25), but a high concentration is toxic. The MAPK pathway might explain the increase in matrix deposition under influence of a low dose of BA in differentiated BMSCs and also the toxicity of a high concentration of B in this study.

Sodium and potassium are electrolytes responsible for membrane polarity and their imbalance can bring about membrane depolarization and the destruction of membrane integrity (26). In the present study, on days 5 and 15 BA caused an increase in sodium uptake and a reduction in the intracellular potassium content. This shows that the balance of these two electrolytes has been well preserved by BA, with the exception of day 10 where the concentration remained unchanged. The explanation of this phenomenon is difficult as we could not find any other report or supporting data, but we hypothesize that at this stage more sodium is needed to initiate mineralization, as in the control group the process of matrix precipitation normally started on day 10. However, we still recommend more investigation be carried out regarding this observation.

In the present study, treatment of the cells with BA caused the activity of ALT and AST to reduce significantly on days 10, 15 and 21but not on day 5. Transaminase enzymes are important in amino acid metabolism, which is key to protein synthesis during development and differentiation of a cell. During osteogenic differentiation 8 groups of proteins are involved, of which the largest category are the enzymes responsible for metabolic activity, such as the Krebs cycle, amino acid metabolism, protein synthesis and glycolysis (27). According to our findings, in the osteogenic period (from day 5 to day 21) activity of ALT and AST increased in the control cells. The B treatment from day 10 onwards caused a reduction in the activity of these enzymes. The reason for this might be the switch between sources of energy since the low concentration of B (6 ng/ml) increased LDH activity which might be considered as a marker of changes from aerobic to anaerobic metabolism. The high concentration of B (6 μg/ml) had no effect on LDH activity and caused significant reduction of ALT as well as AST, an effect on metabolism which might result in the observed viability reduction and mortality induction. The latter hypothesis as an explanation of reductions of AST and ALT activity and elevation of LDH requires more efforts and investigations in future.

One important finding of this study was the early initiation of osteogenicity in the treated groups. Based on this study and others (10, 12, 13) osteogenic differentiation starts at day 10, but treatment with 6 ng/ml and 6 μg/ml of B caused a shift to the left in matrix deposition which in the presence of BA begins on day 5. It showed that B may cause the factors involved in osteogenic differentiation (such as genes, proteins and metabolic enzymes participating in energy production) to express earlier than should normally happen.

## Conclusion

Low dose of BA (6 ng/ml) is not toxic and might be a necessary factor for osteogenic differentiation and induction of early matrix deposition in BMSCs. Although more investigation is required, this data might be a window that gives us a view of the future in which B is prescribed to prevent or cure bone-related complications, such as osteoporosis and bone fracture healing. As the B content of dried fruits, legumes, nuts and avocado ranges from 2 to 4.3 mg/100 g, based on this study and others, the consumption of these foods are highly recommended in cities with a high prevalence of bone-related problems and in persons whose bone have been fractured. Since the B effect is very complex we still recommend more research to be conducted to fully understand the exact mechanism of B in the osteogenic processes of bone.
